# Supplementary material for: Ancient and methane-derived carbon subsidizes contemporary food webs
Source: Nat Commun. 2016 Nov 8;7:13163. doi: 10.1038/ncomms13163 (PMC5117835; doi:10.1038/ncomms13163)
Supplement: Supplementary Information — Supplementary Figures 1-6 and Supplementary Tables 1-4. [file ncomms13163-s1.pdf]

a

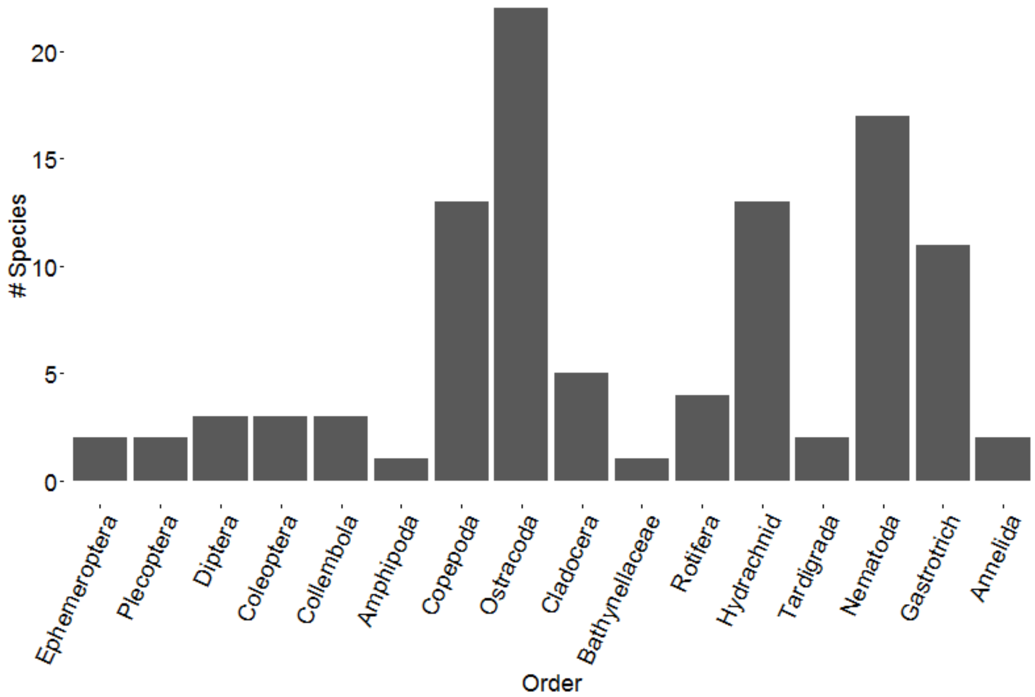

b

Plecoptera Species	Duration larval stage	Wells/locations where common	Diet
Isocapnia crinita	2 years	HA02, HA07, HA10, HA15, HA17	Grazers
Isocapnia grandis	2 years	HA07, HA10, HA15, HA17, Kalispell, Methow	Grazers
Isocapnia integra	2 years	HA02	Grazers
Paraperla frontalis	2-3 years	All Nyack wells, all floodplains	Omnivorous
Kathroperla perdita	2-3 years	All Nyack wells, all floodplains	Omnivorous

Supplementary Figure 1 | Species documented on the Nyack Floodplain A. A total of 104 species have been documented in the hyporheic zone of the Nyack floodplain. Seventeen are Plecoptera, but only 5 Plecoptera species were commonly occurred in well samples (Table 1) and they do not occur in the river channel, spending the entire larval life history in the aquifer. Gibert et al. (*10*), described this novel life history strategy as amphibitic – hatching and growth to larval maturity occur in the aquifer, while adult emergence, mating and egg deposition are focused in the river channel. B. Descriptions and life history characteristics of the five common amphibitic (larval stage underground, adult emergence aboveground) Plecoptera species which were used in our analysis (*8*, *10*, *14*, *41*, *42*, *48*).

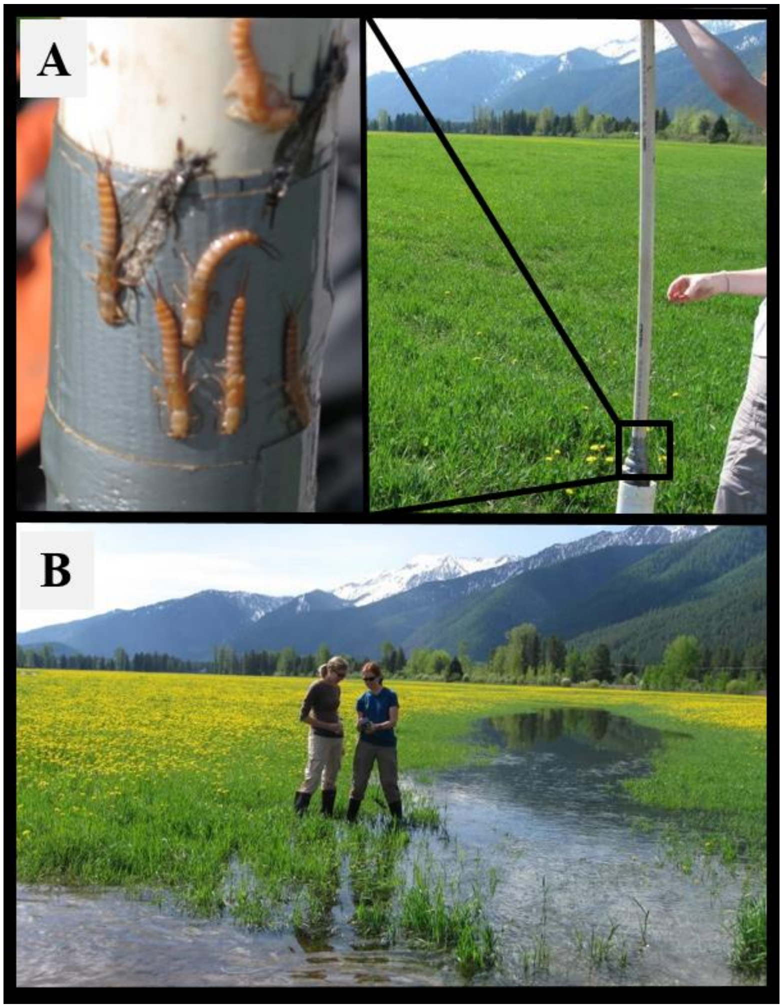

Supplementary Figure 2 | Surface and ground water connectivity A. Large (~2.5 cm) stoneflies (*Paraperla frontalis*) perched on equipment partially removed from well HA05 (Fig 1B) located 1.5 km from the river channel at Nyack. B. Aquifer water emerging at the surface of floodplain in a paleochannel located near the well in A during spring runoff, illustrating the ground and surface water connectivity characteristic of alluvial floodplains like the Nyack. Photos in A are a courtesy of Dr. Ashley Helton.

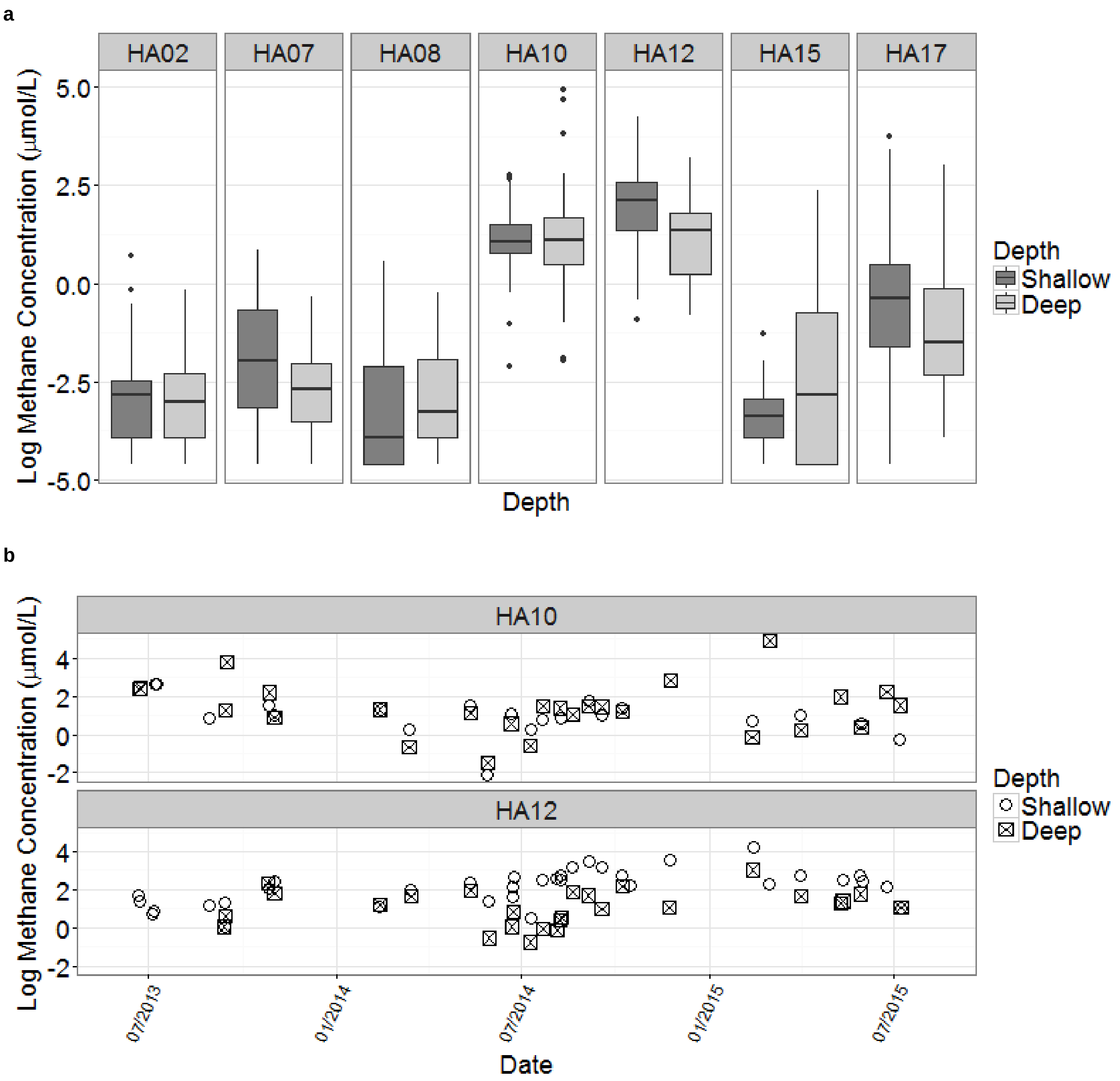

Supplementary Figure 3 | Methane concentrations in the Nyack aquifer A. Boxplots of log-transformed methane concentrations stratified per month show low (<1 µmol/L) methane concentrations in most wells sampled, with significant effects of depth (indicated by the star) occurring at HA10 and HA12. In HA12, shallow methane samples tended to have higher methane concentrations, whereas in HA10 deeper samples tended to have higher methane concentrations, displaying vertical heterogeneity within the aquifer. B. Methane concentrations are plotted by date sampled from February 2014 to September 2015 (average error <0.17 µmol/L). Deeper HA10 samples show erratic changes in concentration over time.

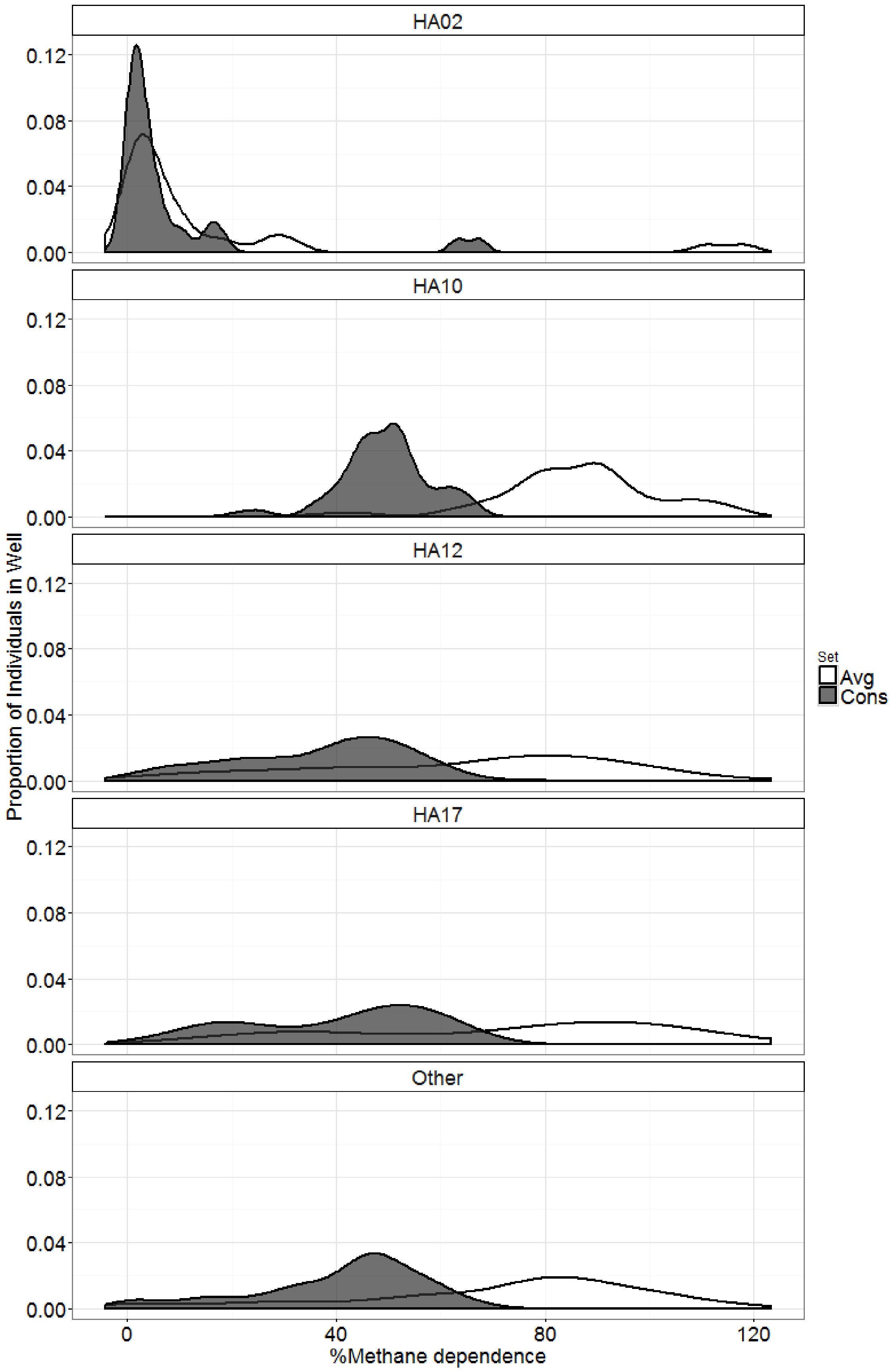
HA02
HA10
HA12
HA17
Other
0.12
0.08
0.04
0.00
0
40
80
120
%Methane dependence
Proportion of Individuals in Well
Set
Avg
Cons

Supplementary Figure 4 | Methane derived carbon contribution across the Nyack aquifer
Average and conservative methane carbon contribution estimates are displayed for focal wells and all other wells ("Other") analyzed in the study. Well HA02, with much lower methane dependence, was closest to the river and had the shortest residence time (45 days). The rest of the wells were all at similar residence times 117 – 304 days (*17*) (Supplementary Table 2). Wells HA10 and HA12 both have high levels of measurable methane. Despite these being the only wells with measurable methane, all wells but HA02 showed 40 to 80% methane dependence as measured by stonefly biomass.

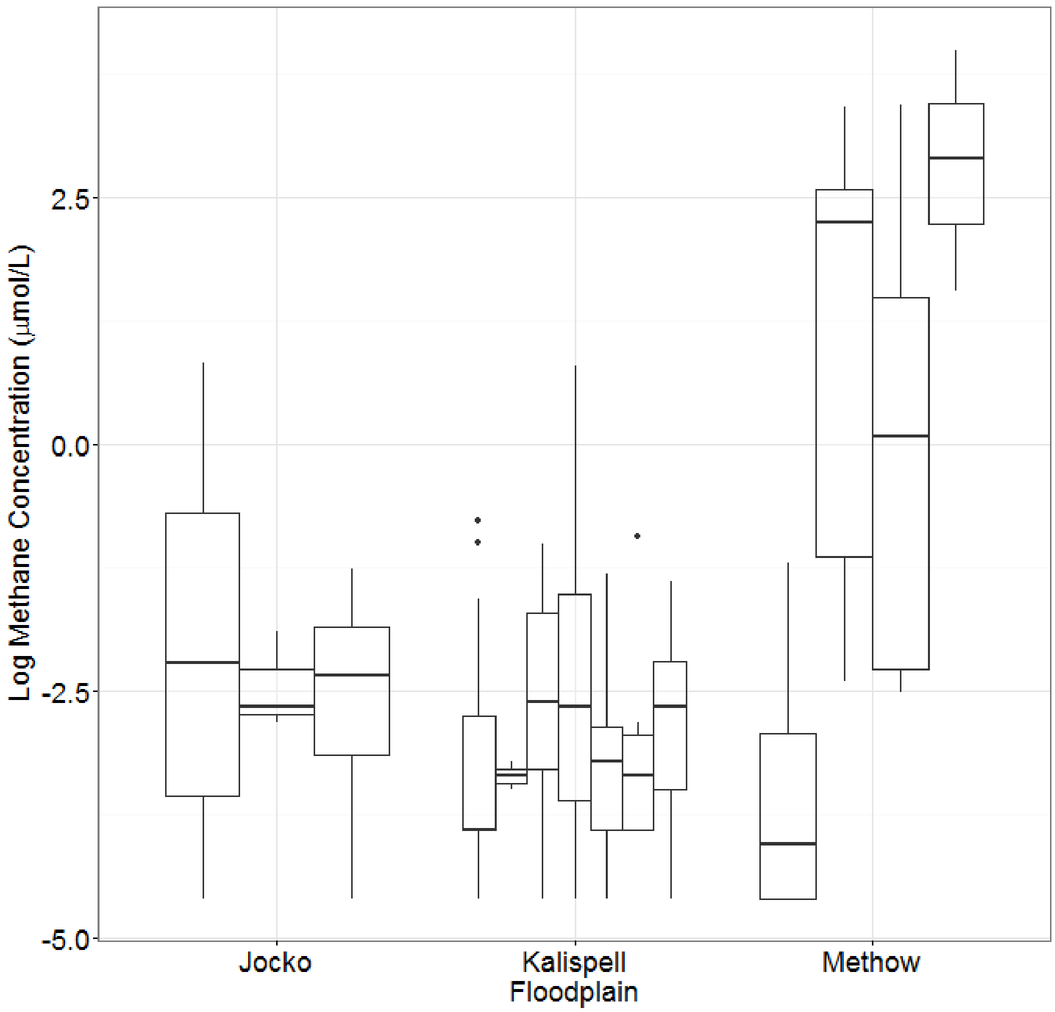

Supplementary Figure 5 | Methane concentrations at other floodplains Log methane concentrations for wells at all other floodplains studied. Most wells had less than 1 μmol/L dissolved methane over the course of study despite producing stoneflies which were clearly dependent on methane-derived carbon.

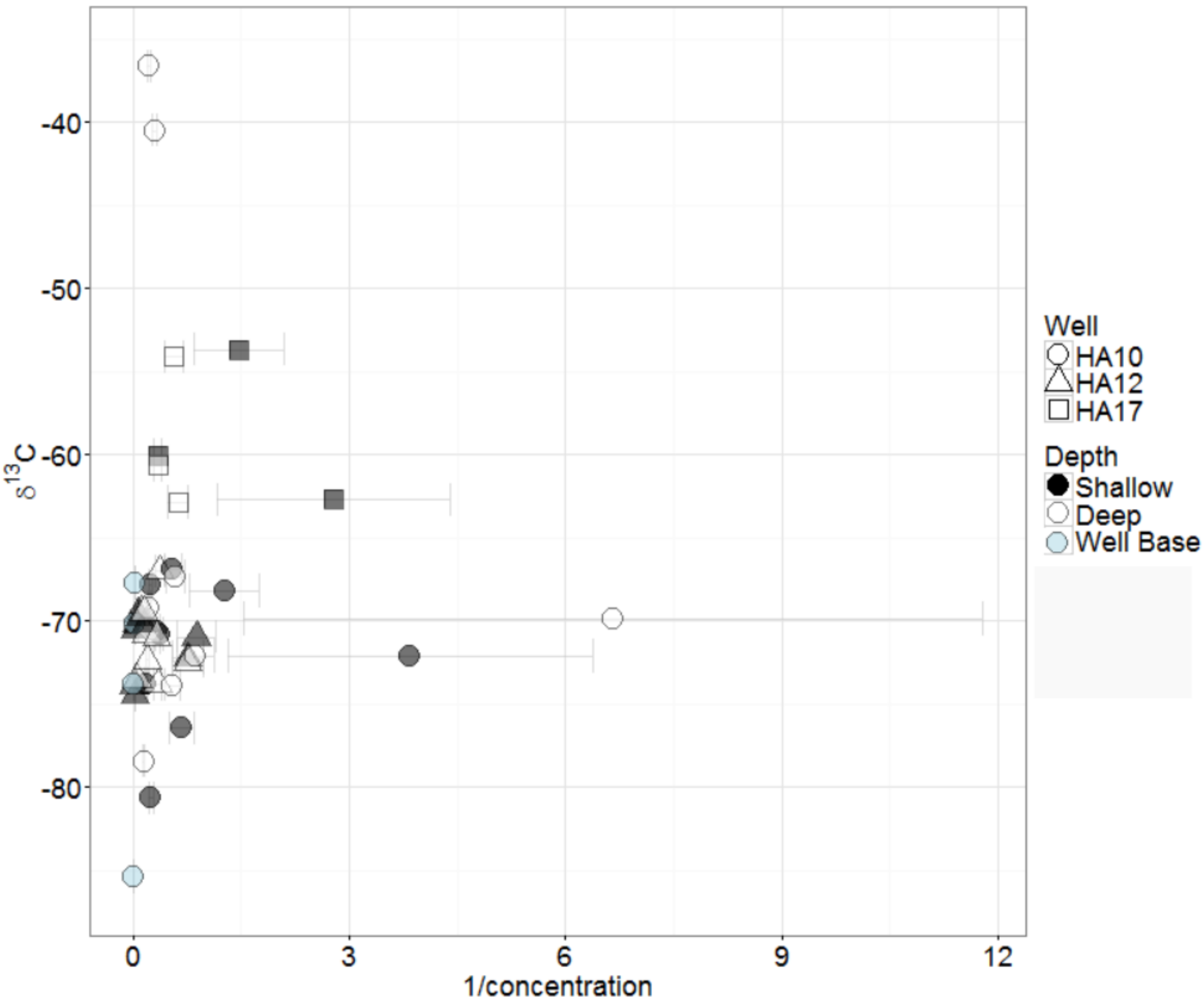

Supplementary Figure 6 | Using a Keeling Plot to determine methane source values A Keeling plot to indicate source $\delta^{13}C$ values of methane that shows a broad range of variation in $\delta^{13}C$ even during periods of high dissolved methane concentrations in the aquifer. We used these relations to parameterize the mixing models using two extreme estimates of source $\delta^{13}C$: the average of $\delta^{13}C$ values in methane samples taken at > 1 µmol/L concentration, and this average fractionated by $\alpha = 30.3$ ‰ (*25*).

Supplementary Table 1 | Nyack well characteristics Well residence times and typical dissolved oxygen concentrations are displayed alongside species which we typically observed in each well. Note that we only found methane in wells with at least rare hypoxia.

Well	Easting	Northing	Shallow RT*	Deep RT*	DO character	Observational Notes
HA02	292244	5369912	45	60	Oxic	Near river channel at head of floodplain
HA07	290489	5372413	156	217	Oxic	
HA08	290564	5372617	210	263	Oxic	
HA10	290586	5373203	117	146	Occasional hypoxia	Methane usually present
HA12	292484	5370507	119	179	Occasional hypoxia	Methane usually present
HA15	291560	5371559	133	210	Oxic	
HA17	291846	5371524	167	304	Rare hypoxia	Rare hypoxia in winter, occasionally has methane in low concentrations

*RT = residence time (days). Estimates taken from Helton et al. (2012) (*17*).

Supplementary Table 2 | Radiocarbon-aged methane sample metadata Sample information for all methane samples which were radiocarbon aged. Ancient methane contribution estimates were calculated using the same two-source mixing model equation used for stonefly overall methane contributions, but using radiocarbon signatures and defining ancient methane as radiocarbon-dead. Sample sizes were limited because a) we needed to meet minimum methane concentrations for analysis, and b) sample radiocarbon dating was very cost-prohibitive. Using this data, it is impossible to determine the maximum methane age, especially at HA10, and also to determine the depth at which methane is generated. However, as shown in Supplementary Figure 2B, methane concentrations tended to be higher at the deeper depth in HA10. Well sample depths are indicated as following: S=shallow, D=deep, and WB=well base.

Date	Well and Depth	Methane $\delta^{13}C$ (‰)	Methane $\Delta^{14}C$ (‰)	Methane age (years BP)	Methane concentration (µmol/L)	CO_2 $\delta^{13}C$ (‰)	CO_2 $\Delta^{14}C$ (‰)	CO_2 age (years BP)	Ancient methane contribution
9/4/2014	HA12 S	-70.49 ± 0.3	-122.6 ± 21.0	990 ± 200	21.99 ± 0.24	-15.7 ± 0.15	-160.4 ± 1.8	1340 ± 20	12.3 %
9/17/2014	HA12 S	-70.16 ± 0.3	-92.8 ± 19.7	720 ± 180	28.39 ± 0.24	-14.8 ± 0.15	-181.0 ± 4 1.	1545 ± 20	9.2 %
11/24/2014	HA12 S	-72.60 ± 0.15	-48.2 ± 1.7	335 ± 15	30.83 ± 0.24	-19.2 ± .15	-158.4 ± 1.7	1325 ± 20	0.5 %
11/24/2014	HA10 S	-70.61 ± 0.3	-580.0 ± 7.2	6910 ± 140	2.80 ± 0.24	-16.8 ± 0.15	-223.4 ± 1.5	1970 ± 20	58.0 %
5/12/2015	HA10 D	-70.76 ± 0.3	-328.5 ± 1.3	3200 ± 20	1.80 ± 0.09	-15.2 ± 0.15	-150.3 ± 1.5	1310 ± 15	32.8 %
8/16/2015	HA10 WB	-84.40 ± 0.15	-253.7 ± 1.3	2350 ± 15	4.92 ± 0.09	-14.9 ± 0.15	-217.2 ± 1.4	1965 ± 15	25.4 %

Supplementary Table 3 | Bayesian modeling scenarios We used four scenarios to represent extremes of two ranges: methane $\delta^{13}C$ and ancient methane $\Delta^{14}C$. These scenarios incorporated the displayed set of values used in parameterizing the Bayesian mixing model. Please mote the differences in OM radiocarbon values; these resulted from varying methane $\delta^{13}C$ values used to calculate the weighted average of OM $\Delta^{14}C$ (weighted by non-methane based biomass). See methods for more information.

**Scenario**	**ConsAge**		**AvgAge**		**ConsAnc**		**AvgAnc**	
**α**	-30.3 ‰		0		-30.3 ‰		0	
**Methanogenic methane max age**	Measured (6900 yrs BP)		Measured (6900 yrs BP)		Radiocarbon-dead (>50,000 yrs BP)		Radiocarbon-dead (>50,000 yrs BP)	
	$\delta^{13}C$	$\Delta^{14}C$	$\delta^{13}C$	$\Delta^{14}C$	$\delta^{13}C$	$\Delta^{14}C$	$\delta^{13}C$	$\Delta^{14}C$
**Modern methane**	-100.8 ± 8.5	0 ± 7.2	-68.8 ± 8.5	0 ± 7.2	-100.8 ± 8.5	0 ± 7.2	-68.8 ± 8.5	0 ± 7.2
**Aged-Ancient methane**	-100.8 ± 8.5	-580 ± 7.2	-68.8 ± 8.5	-580 ± 7.2	-100.8 ± 8.5	-1000 ± 7.2	-68.8 ± 8.5	-1000 ± 7.2
**Organic matter**	-27.8 ± 2.5	-65.6 ± 75.9	-27.8 ± 2.5	-13.7 ± 32.2	-27.8 ± 2.5	-65.6 ± 75.9	-27.8 ± 2.5	-13.7 ± 32.2

Supplementary Table 4 | Organic matter pools Mean and standard error values for organic matter pools as measured during July 2013. CPOM (Coarse particulate organic matter) incorporated some stonefly detritus, resulting in its low value. FPOM stands for fine particulate organic matter. The average and standard error were incorporated into the Bayesian mixing model.

OM Type	$\delta^{13}C$	Std. Deviation	n
Biofilm	-28.50	1.81	26
CPOM	-29.71	3.56	28
FPOM	-25.28	1.65	24
**Average**	**-27.83**	**2.49**	
